# Bioprospecting endophytic fungi for bioactive metabolites with seed germination promoting potentials

**DOI:** 10.1186/s12866-024-03337-x

**Published:** 2024-06-08

**Authors:** Dina El-Nagar, S. H. Salem, Fatma I. El-Zamik, Howaida M. I. Abd El-Basit, Y. G. M. Galal, SM Soliman, HA Abdel Aziz, M. A. Rizk, El-Sayed R. El-Sayed

**Affiliations:** 1 Soil and Water Research Department, Nuclear Research Center, Egyptian Atomic Energy Authority, Cairo, Egypt; 2 Department of Microbiology, Faculty of Agriculture, Zagazig University, Zagazig, Egypt; 3 Plant Research Department, Nuclear Research Center, Egyptian Atomic Energy Authority, Cairo, Egypt

**Keywords:** Seed germination, Antioxidant, Antifungal, Endophytes, Plant growth promotion

## Abstract

There is an urgent need for new bioactive molecules with unique mechanisms of action and chemistry to address the issue of incorrect use of chemical fertilizers and pesticides, which hurts both the environment and the health of humans. In light of this, research was done for this work to isolate, identify, and evaluate the germination-promoting potential of various plant species’ fungal endophytes. *Zea mays* L. (maize) seed germination was examined using spore suspension of 75 different endophytic strains that were identified. Three promising strains were identified through screening to possess the ability mentioned above. These strains *Alternaria alternate, Aspergilus flavus*, and *Aspergillus terreus* were isolated from the stem of *Tecoma stans*, *Delonix regia*, and *Ricinus communis*, respectively. The ability of the three endophytic fungal strains to produce siderophore and indole acetic acid (IAA) was also examined. Compared to both *Aspergillus flavus* as well as *Aspergillus terreus*, *Alternaria alternata* recorded the greatest rates of IAA, according to the data that was gathered. On CAS agar versus blue media, all three strains failed to produce siderophores. Moreover, the antioxidant and antifungal potentials of extracts from these fungi were tested against different plant pathogens. The obtained results indicated the antioxidant and antifungal activities of the three fungal strains. GC-Mass studies were carried out to determine the principal components in extracts of all three strains of fungi. The three strains’ fungus extracts included both well-known and previously unidentified bioactive compounds. These results may aid in the development of novel plant growth promoters by suggesting three different fungal strains as sources of compounds that may improve seed germination. According to the study that has been given, as unexplored sources of bioactive compounds, fungal endophytes have great potential.

## Key points

• Discovery of three promising fungal endophytes with seed germination promoting potential.

• Indole acetic acid and siderophores production were tested for the three strains.

• Extracts of the three strains showed antifungal and antioxidant activities.

• Bioactive chemical constituents of the three fungi were analyzed by GC-MS.

## Introduction

Natural bioactive chemicals are regarded as a critical component in the production of commodities of great value. Their biological activity has enabled their usage in the fields of agriculture, medicine, and the nutritional sector [1]. Two of the most important and challenging scientific undertakings are the search for new bioactive chemicals and the assessment of their ability to have biological impacts [2]. For example, the issue of inappropriate and irresponsible insecticide and chemical fertilizer usage, which hurts both the natural world and the health of people calls for the continual development of new natural chemicals. In addition, resistant plant-infecting bacteria have appeared [3]. The health of humans and animals could be seriously harmed by the resistant microorganisms. Therefore, it is more important than ever to find and create new natural substances to satisfy this pressing and expanding need. Utilizing microbial communities, particularly fungus, provides a number of benefits that make it more effective than alternative tactics. Due to their numerous biotechnological applications, as well as their affordability and ease of use as a culture medium for, say, the development and metabolism of fungi, cells have garnered a lot of interest [4]. In an effort to discover new compounds with a range of functions, recent research has concentrated on bioprospecting endophytic fungi, or the fungi that reside inside plant tissues [5–7]. Endophytic fungi have mainly been examined as producers of innovative bioactive substances and additional metabolic products from plant hosts [8–10]. It was demonstrated that these substances were untapped sources of new bioactive compounds [11–13].

Endophytic fungi are microorganisms that can boost agricultural output and occupy plant tissues and organs without exhibiting any signs of harm. The increased availability of nutrients (nitrogen, phosphorus, potassium, zinc, iron, etc.) and the creation of plant hormones are some of the ways in which endophytic fungi promote plant development [14]. The development of endophytic fungus on roots, according to Rodriguez et al. [15] and Redman et al. [16], can affect soil structure, plant hormone balance, the chemical constituents of exudates from roots, plant hormone balance, and plant defense against both biotic and abiotic stimuli. In addition to having significant biotechnological potential (producing enzymes, biocleaning, biotransformation, etc.) [17]. The diverse collection of endophytes is found in the roots and/or shoots of the plant. These have the capacity to lessen the host’s vulnerability to biotic and abiotic challenges, such as heat, salt, and drought stress [16]. Depending on the fungal strain, host genotype, and growing circumstances, endophytes exhibit a wide range of functional variety, from pathogenicity to mutualism [18].

Endophytic fungi, which live inside the tissues of plants and help in the absorption of nutrients, are currently thought to exist in the majority of plants, the creation of chemicals that promote plant development, and the production of antimicrobials that are crucial for the survival of plants [19]. Additionally, compared to their free counterparts, they displayed increased metabolic activity [20–25]. Due to all of these factors, we aim to examine in this work the unexplored potential of endophytic fungi from multiple species of plants as sources of compounds with the potential to improve *Zea mays* L. (maize) seeds in germination.

## Materials and methods

### Plant samples and isolation of fungal endophytes

Several leaf and stem samples were obtained from healthy plants in various areas around Egypt. The endophytic fungus was isolated using 26 distinct species of plant types. (Table 1). The samples were procured in sterile polythene bags and delivered to the lab, where they were processed within 24 h and stored at 4 °C for later use.

**Table 1:** Host plants, isolated endophytic fungi, and effects of their chloroform extracts on germination of *Zea mays* L. seeds

Host Plant	Number of fungi/sample		Total number	Fungi with germination potential	Germination percentage (%)	
	from leaves	from stems			From leaves	From stems
*Triticum L.*	2	4	6	2	6.67 ± 0.34^h^	30.00 ± 11.56
*Trifolium L.*	1	1	2	2	20.00 ± 1.78^f^	10.0 ± 0.78^m^
*Chorisia crispifolia*	2	1	3	2	6.67 ± 0.34^h^	53.33 ± 12.03^gh^
*Medicago sativa*	3	3	6	2	26.67 ± 2.83^e^	6.67 ± 0.67^mn^
*Cupressus macrocarpa*	2	3	5	2	33.33 ± 4.55^d^	6.67 ± 0.34^mn^
*Delonix regia*	1	3	4	1		86.67 ± 18.83^c^
*Morus alba*	0	1	1	1		13.33 ± 8.83^lm^
*Paulownia elongata*	4	1	5	2	23.33 ± 1.83^ef^	16.67 ± 4.83^k^
*Tecoma orange*	1	2	3	2	10.00 ± 0.78^g^	53.33 ± 14.55^gh^
*Duranta L.*	1	1	2	0		
*Citrus x sinensis*	2	0	2	1	20.00 ± 0.78^f^	
*Mangifcra indica*	3	0	3	1	33.33 ± 4.83^d^	
*Psidium guajava*	0	3	3	1		80.0 ± 11.56^d^
*Ocimum basilicum*	2	1	3	2	73.33 ± 14.55^a^	66.67 ± 8.83^f^
*Cassia fistula*	2	0	2	1	20.00 ± 5.78^f^	
*Tecoma stans*	2	3	5	2	3.33 ± 0.34^h^	96.67 ± 3.34^a^
*Nerium oleander*	3	1	4	2	50.00 ± 5.29^b^	40.0 ± 11.56^j^
*Hibiscus L.*	0	1	1	0		
*Ricinus communis*	3	2	5	2	70.00 ± 11.56^a^	90.0 ± 5.78^b^
*Salvia officinalis*	1	2	3	2	43.33 ± 4.07^c^	56.67 ± 17.66^g^
*Prunus domestica*	0	1	1	1		6.67 ± 0.34^mn^
*Rosmarinus officinalis*	0	1	1	1		3.33 ± 0.34^n^
*Echinacea purpurea*	1	1	2	2	36.67 ± 4.55^d^	70.00 ± 15.29^e^
*Melissa officinalis*	0	1	1	1		3.33 ± 0.34^n^
*Terminalia arjuna*	1	0	1	1	70.00 ± 11.56^a^	
*Malus domestica*	0	1	1	1		45.67 ± 8.83^i^
Control					50.00 ± 5.78^b^	50.00 ± 5.78^h^
Total	37	38	75	37		

PDA was used for endophytic fungi isolation. The calculated mean is for triplicate measurements from two independent experiments ± SD, ^a−n^ means with different superscripts in the same column for each nanoparticle are considered statistically different (LSD test, *P* ≤ 0.05)

Following Ismaiel et al. [26] technique, each plant species had an endophytic fungus isolated. Plant materials (leaf and stem) were subjected to a process of surface sterilization that started with a thorough washing under running water for five minutes to remove dust and debris, followed by air drying, immersion in 70% ethanol for one minute, one minute of submersion in 5% solution of sodium hypochlorite (NaOCl), draining, and immersion in 70% ethanol for 30 s. After being cut into tiny (2–3 cm) pieces under sterile conditions, these specimens were washed 4–6 times with sterilized distilled water in the final. On the surface of the samples, sterile filter paper was used to dry them [27]. Slices of the plant material, each measuring 0.5 cm by 0.5 cm, were placed on Petri dishes with potato dextrose agar (PDA) medium (contains potato 200 g L^− 1^, dextrose 20 g L^− 1^, and agar 15 g L^− 1^, pH6.0) with antibiotic streptomycin sulfate (250 g ml^− 1^, Sigma) at 28 °C until fungal growth was started. Throughout this time, the plates were meticulously covered in parafilm to prevent contamination and desiccation of the medium.

### Purification and preservation of endophytic fungi

Hyphal tips were put on new PDA plates without antibiotics in order to isolate endophytic fungi from plant tissue (from master plates). After many days of incubation, each fungal culture’s purity was evaluated by gauging the colony’s form. Up until pure cultures for identification were achieved, continuous plates were sub-cultured. Under a light microscope, endophytic strains were morphologically identified and analyzed (Olympus, USA). After being properly labeled and transferred individually to PDA slants, the purified endophytic isolates were stored at 4ºC until use.

### Screening the isolated endophytes for their seed germination promoting potential

Maize *(Zea mays* L.), the third most important food crop globally after wheat and rice, was chosen as the test model. The seeds of *Zea mays* L. were surface sterilized for 5 min with 70% ethanol (v/v) and 10 min with 5% sodium hypochlorite, followed by several rinses with sterile distilled water. Ten healthy, surface-sterilized seeds were then inserted in 20 ml of each isolate’s endophytic fungus spore suspension (two-week-old fungal culture) and shaken for 24 h. The control consisted of a flask containing the same volume of sterile water. The soaked seeds were next put onto two layers of filter paper (Whatman # 1, size 90 mm) in autoclaved Petri dishes (90 × 15 mm), with a total of 10 seeds per Petri dish. There were three duplicates of each treatment. All of the seeds were cultivated in a chamber for growth at a temperature of 28 °C with sterile distilled water at regular intervals. After 10 days of incubation, the plates were checked daily, and the germination percentage for both the fungal and control treatments (sterile distilled water and culture media) was determined. The seeds were considered to have germinated when the sprouting radicles measured longer than 0.5 cm. The germination percentage is computed by applying the following equation:

$$\begin{array}{l}{\rm{\% }}\,{\rm{Of}}\,{\rm{Seed}}\,{\rm{germination}}\,{\rm{ = }}\,\\\left( {{\rm{Number}}\,{\rm{of}}\,{\rm{germinated}}\,{\rm{seeds}}\,{\rm{/}}\,{\rm{Total}}\,{\rm{number}}\,{\rm{of}}\,{\rm{seeds}}} \right)\,{\rm{100}}\end{array}$$

### Fungal strains

As previously mentioned, the ability of 75 endophytic fungus isolates to promote seed germination was evaluated. Three different isolates of spore suspension from the separated fungi were discovered to have the ability to promote seed germination. These strains included *Alternaria alternata, Aspergillus flavus*, and *Aspergillus terreus*, all of which were isolated from the stems of *Tecoma stans, Delonix regia*, and *Ricinus communis*, respectively. The three distinct strains were located and assigned the numbers AUMC15170, AUMC15171, and AUMC15172 in the Assiut University Mycological Centre’s Culture Collections (aun.edu.eg/aumc/aumc.htm).

### Identification of the selected endophytic fungi

The three strains of isolated endophytic fungi were identified using both morphological and molecular techniques. Using the technique as explained by Qadri et al. [28] morphological identification was completed. Using the Culture Slide Technique, semi-permanent slides were created from cultures, stained with Lactophenol Cotton Blue (LPCB), and studied at 40- and 100-times magnifications to characterize the morphology of fungal isolates.

#### Fungal isolate molecular identification

The isolates were cultivated on sterile Petri plates with autoclaved Czapeks yeast extract agar (CYA) for species of *Aspergillus* and V8 Juice for species of *Alternaria*, and then grown for seven days in an incubator at 28 °C [29]. The growing cultures were delivered to the Molecular Biology Research Unit at Assiut University, where they were used to extract DNA using the Patho-gene-spin DNA/RNA extracting kits provided by the Korean Intron Biotechnology Company. Following that, the fungal DNA was sent to SolGent Company in Daejeon, South Korea, for rRNA gene sequencing as well as polymerase chain reaction (PCR). The reaction mixture for PCR contained the primers ITS1 (forward) and ITS4 (reverse). Both ITS1 (5’-TCC GTA GGT GAA CCT GCG G-3’) and ITS4 (5’-TCC GCT TGA TAT GC-3’) are used. Using the identical primers and ddNTPs added to the reaction mixture, Sequencing of the amplicons from the purified PCR product was done [30]. using the National Centre for Biotechnology Information (NCBI) website’s Basic Local Alignment Search Tool, the collected sequences were examined (BLAST). The sequences were phylogenetically analyzed using MegAlign (DNA Star) software version 5.05. The sequence sizes of *Alternaria alternata* (AUMC15170), *Aspergillus terreus* (AUMC15172), *Aspergillus flavus* (AUMC15171) were successfully deposited in NCBI GenBank with accession numbers OR511885 (https://www.ncbi.nlm.nih.gov/nuccore/OR511885), OR511886 (https://www.ncbi.nlm.nih.gov/nuccore/OR511886), and OR511887 (https://www.ncbi.nlm.nih.gov/nuccore/OR511887), respectively.

### Cultivation conditions and preparation of fungal culture extracts

According to Nurunnabi et al. [31], the secondary metabolites were extracted. The fungal isolate was cultured in conical flasks (250 mL) using 50 mL potato dextrose broth (PDB) at 28 °C for 15 days. The filtrates were subsequently extracted three times with the same amount of the solvent methylene chloride in a separating funnel after the mycelium had been removed from the culture broth with a filter (Whatman® qualitative filter papers, Grade 1; Sigma-Aldrich, USA). Anhydrous sodium sulfate was employed to pool and separate the layers of methylene chloride, followed by water removal. A German IKA, RV10 rotary evaporator was used to concentrate the methylene chloride layers through evaporation at low pressure. After being thoroughly dissolved in methanol of HPLC-grade, the resultant dry film was examined.

### Indole acetic acid production

Each fungal strain was separately inoculation into a 250 ml Erlenmeyer conical flask with 50 ml potato dextrose broth medium (PDB), which was enhanced with a precursor of 0.4% tryptophan. The inoculated flasks were subsequently grown for 14 days at 30 °C depending on agitation condition (150 rpm). Following incubation, the cell-free supernatants should be separated. The fungus was spun for 10 min at 10,000 rpm, which were then employed as sources of indole acetic acid (IAA). Indole acetic acid assay: The reaction mixture comprised 2 ml of Salkowski’s reagent (one ml of 0.5 M FeCl3 as diluted into 50 ml from 35% perchloric acid) and 1 mL of cell-free supernatant. After that, the entire mixture was incubated at 28 °C for 30 min. At 530 nm, calorimetric quantification was performed as compared with the IAA standard curve [32].

### Quantification of phosphate solubilization in submerged fermentation

The tested strains (AUMC 15,170, AUMC 15,171, and AUMC 15,172) were inoculated separately in 250-ml Erlenmeyer conical flasks each containing 50 ml of PVK broth medium (pH 7). The PVK medium contained: calcium phosphate (5.0 g/L), ammonium phosphate (5.0 g/L), glucose (10.0 g/L), yeast extract (5.0 g/L), potassium chloride (0.2 g/L), magnesium sulphate (0.1 g/L), manganese sulphate (0.001), and ferrous sulphate (0.001) The final pH was adjusted to 7.0. Each flask received 1.0 mL of spore suspension from a 7-day-old culture and contained 1.5 × 10^8^ spore/mL. The flasks were then incubated at 30 °C for 10 days in agitated conditions at 150 rpm. After the incubation period, the flask’s contents were filtered and the cell-free supernatants were obtained after centrifugation at 10,000 rpm for 10 min.

#### Assay of phosphate-solubilizing efficiency

The reaction mixture included 100 µL of the supernatant, 10 mL of chloromolybdic reagent (15 g of ammonium molybdate in 400 mL distilled water and 342 ml of concentrated HCl, and the total volume was made up to 1 L.). The reaction mixture was well shaken before being diluted with distilled water to a final volume of 40 mL. 5 drops of the chlorostannous acid reagent (10 g of SnCl2.2H2O diluted in 25 ml of strong HCl) were added along the side and thoroughly mixed. The final volume was completed to 50 mL with distilled water. The absorbance of the obtained blue color was measured at 660 nm against the blank. The concentration of the soluble phosphate was calculated using potassium dihydrogen phosphate (KH_2_PO_4_) as standard. The amount of soluble phosphate was expressed as µg/mL.

### Antioxidant activity

Thaipong et al. [33] used the 2,2-diphenyl picrylhydrazyl (DPPH, Sigma-Aldrich, St. Louis, MO, USA) scavenge free radicals’ experiment to assess the antioxidant properties of the three fungal isolates. Simultaneously, ascorbic acid (Sigma-Aldrich, St. Louis, Missouri, USA) was tested as the positive control. A difference in absorbance between the combination (DPPH + fungal crude extracts) and DPPH solution alone (control) was used to assess the scavenging activity (%).

### Antifungal sensitivity tests

The possible antifungal effects of the extracts were investigated using agar-well diffusion method, in accordance with Pongtharangkul and Demirci [34]. The antifungal test targeted the three plant infections *Penicillium digitatum* AUMC14725, *Botrytis cinerea*, and *Fusarium oxysporium* EUM37, as well as the human pathogen *Aspergillus brasiliensis* ATCC16404. Under the same conditions, nystatin, a common antifungal, and methanol alone were utilized as positive controls. Zones of inhibition were carefully evaluated following incubation.

### GC-MS analysis of the fungal extracts

The three distinct fungus strains’ methanolic extracts were examined using Thermal Scientific’s Trace GC Ultra/ISQ Single Quadrupole MS, TG-5MS fused silicon capillary columns (30 m, 0.251 mm, a 0.1 mm film thickness). Helium was employed as the carrier gas in an electron ionization system with an ionization energy of 70 eV and a constant flow rate of 1 mL/min. The MS transfer line and injector were both heated to 280 °C. To analyze the quantification of all detected components, a percent relative peak area was utilized. The chemicals were tentatively identified based on a comparison of their retention periods and mass spectra with those from the GC/MS system’s NIST, WILLY library.

### Statistics

The standard deviation and mean from three independent experiments’ triplicate measurements were used to express the recorded data. Statistical significance was determined using the SPSS software (v. 22, IBM, NY) and the analysis of variance (ANOVA) as well as Dunken’s test with 95% confidence intervals.

## Results and discussion

### Isolation and screening of potential fungal endophytes

**Fig. 1:**
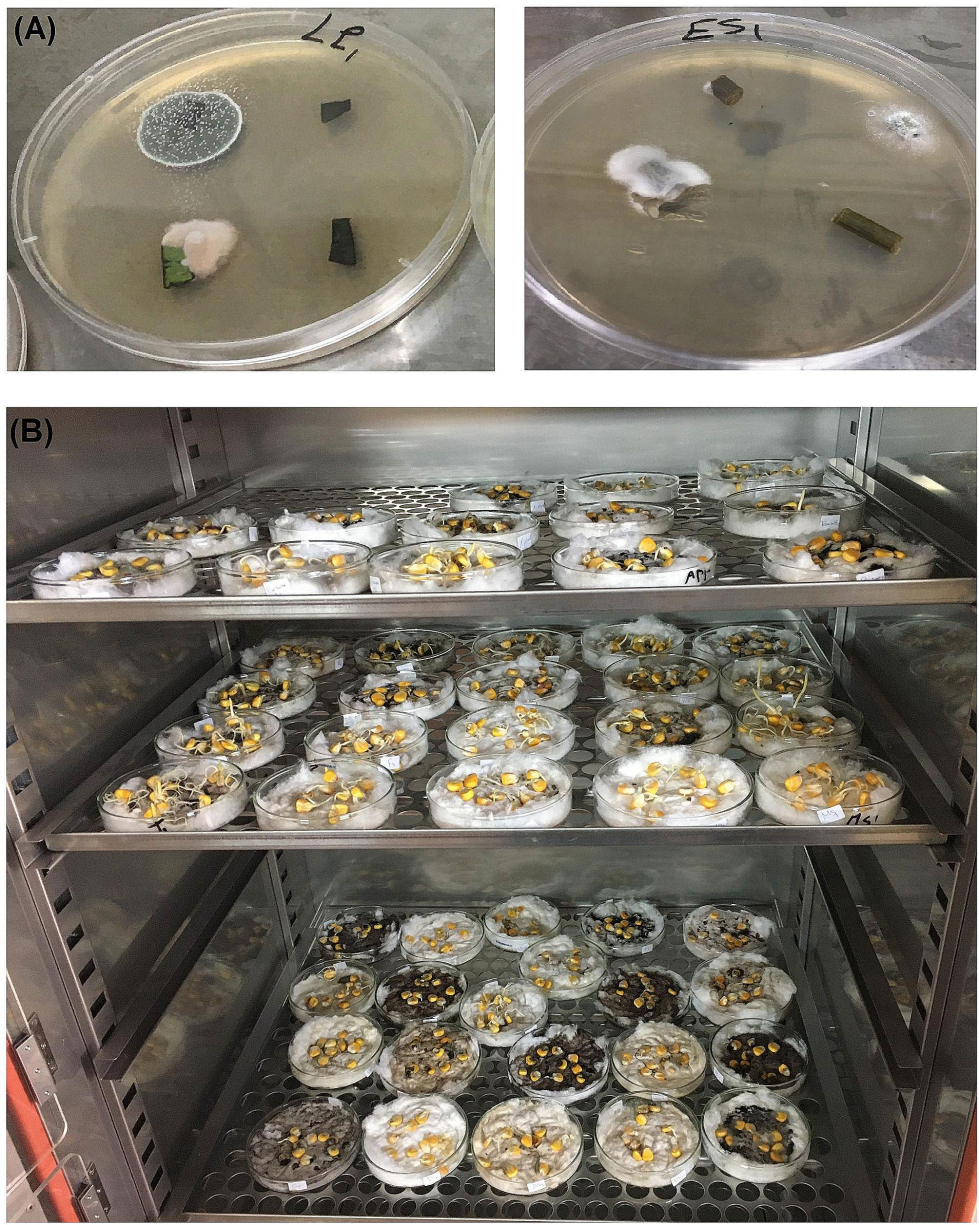
Photographs of endophytic fungi isolation and testing the *Zea mays* L. seeds-promoting germination potential of their chloroform extracts

Endophytic fungi from various plant components, such as leaves and stems, were isolated from 26 distinct plant species (Table 1). 75 endophytic fungi were morphologically distinct and were taken from 26 distinct plant species’ leaves and stem tissues. Stock cultures were kept alive by sub-culturing once a month. After a 7-day growth period at pH 7 and 28 °C, the slants were maintained at 4 °C. According to the study’s findings, which are displayed in Table 1, endophytes were equally common in plant stems and isolated leaves. To create a fungal spore suspension, The 75 isolates were individually grown for 7 days in a potato-dextrose broth at 28 °C. Tween 80 (one drop) was added to sterile water, followed by the addition of the solution to the fungal culture and gentle homogenization. The effects of each spore suspension from the isolated fungus on the germination of maize seeds were examined independently. 37 fungal isolates had different impacts on the germination of maize seeds, according to the screening profile of all the identified endophytic fungi shown in Table 1. It was obvious that the seed germination differs under different endophytic fungi samples (Table 1) where the application of isolated endophytes to maize seeds significantly increased or decreased seed germination compared to controls. Among the 37 fungal isolates, the best three isolates according to the recorded germination percentages were chosen. The seed germination is significantly higher when treated with these three fungal suspensions compared to the treatments with other isolates. Generally, by enhancing seed germination, fungus spore solution pretreatment considerably improved the biological response of the maize seeds (Table 1; Fig. 1). However, compared to the control treatments, endophytic fungi caused a considerable increase in seedling shoot length. The three isolates were thus chosen in order to carry out further identification, testing, and chromatographic characterization of their extracts. Similar outcomes were also reported in the literature by Khan et al. [35], who after six days of incubation observed 100% germination in seeds treated with endophytic. The enhanced germination of maize seeds by endophytes can be used for seed priming processes, which will encourage crop plant development under adverse environmental circumstances. Both isolates of *Aspergillus terreus* (65P and 9 F) have the ability to increase germination in cucumber seedlings by 10 to 20% when grown under controlled circumstances [36]. Out of the fourteen fungal endophytes examined by Lalngaihawmi et al. [37], *Penicillium citrinum* exhibited the highest percentage of rice seed germination (96.65%). Seed priming treatments using endophytes from *Beauveria bassiana*, *Metarhizium anisopliae*, and *Bacillus subtilis* can increase maize seed quality overall [38]. *M*. *anisopliae* showed the greatest increase in germination among them (4.34%). Bio-priming has a significant impact on seed germination, viability, growth, and yields, according to research [39, 40]. Much like how priming enhances seed quality, seedling growth, and crop yield even under adverse conditions. According to Kumar et al. [41], priming with endophytes has also increased disease resistance and tolerance to a range of biotic and abiotic stressors. In general, seed priming with endophytic fungi-based priming could provide better outcomes for the management of crop productivity. Thus, the search for new endophytic fungal species would provide several opportunities such as enhancing the seed characteristics in terms of seed vigor, germination rate, overall crop productivity under changing environmental conditions, and disease resistance and stress tolerance genes which is helpful in simultaneous improvement of seed quality and enhancement in agricultural productivity [41, and references therein].

### Morphological and molecular characterization

**Fig. 2:**
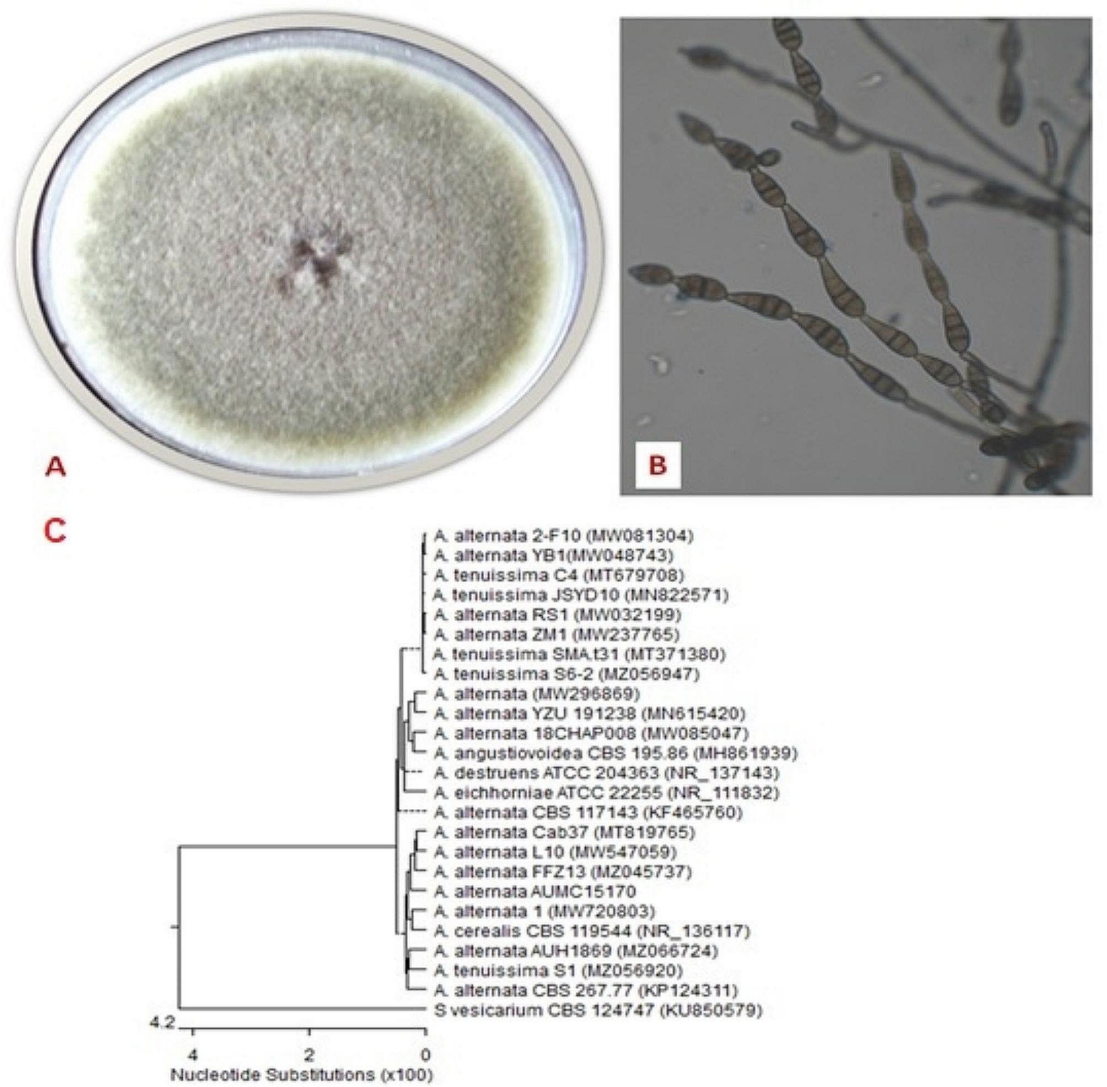
Morphological and molecular characteristics of *A. alternata* AUMC15170. Colony growth was observed on Czapek Yeast autolystae agar after incubation for 10 days at 25 °C (**A**). Microscopic appearance of conidia and conidiophore (**B**). Phylogenetic tree of the fungal isolate (AUMC15170) and other closely related strains of *A. alternata*, based on the ITS1-5.8 S rRNA-ITS2 rDNA sequences **C**)

AUMC15170’s *Alternaria alternative* colony shape is depicted in Fig. 2. A rapidly expanding colony was grown for 10 days at 28 °C on PDA. in a range of black to olivaceous-black or greyish color. When viewed under a microscope, chains of conidia with beaked, obclavate black conidia that each had transverse and longitudinal septa in basipetal succession were visible. The gathered data proved that sample AUMC15170 displayed 99.65–99.83% identification and 98.0–99% coverage with a number of *Alternaria alternata* strains. The morphological criteria for identifying *Alternaria alternata* were identical to those of the fungus that has been cultured for 10 days at 25 °C on CYA agar [29]. As a result, the information gleaned from these strains’ molecular analyses supported the remarkable conformance of these strains to their closely related fungus.

**Fig. 3:**
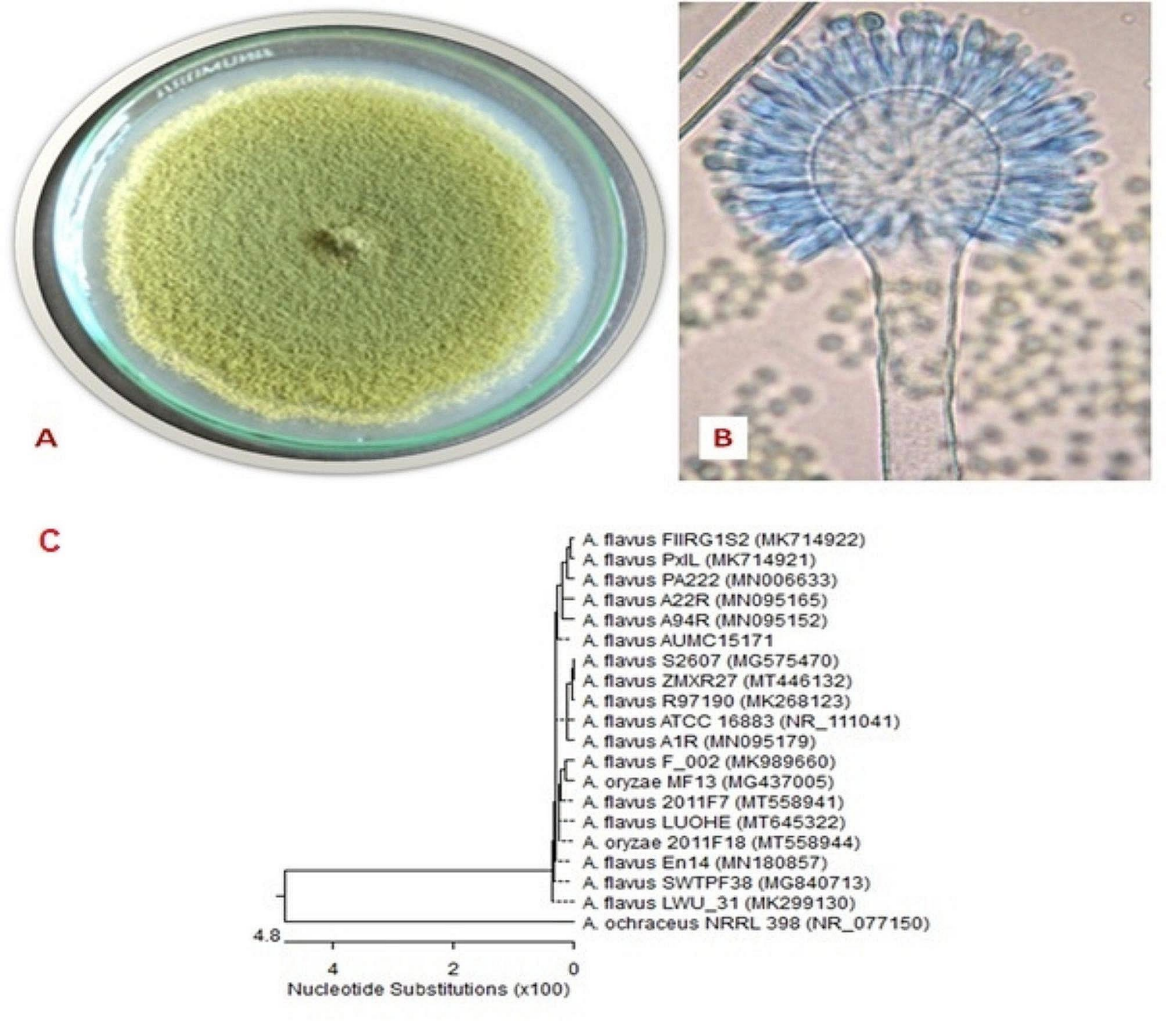
Morphological and molecular characteristics of *A. flavus* AUMC1517. Colony growth was observed on Czapek Yeast autolystae agar after incubation for 10 days at 25 °C (**A**). Microscopic appearance of conidia and conidiophore (**B**). Phylogenetic tree of the fungal isolate (AUMC1517) and other closely related strains of *A. flavus*, based on the ITS1-5.8 S rRNA-ITS2 rDNA sequences (**C**)

**Fig. 4:**
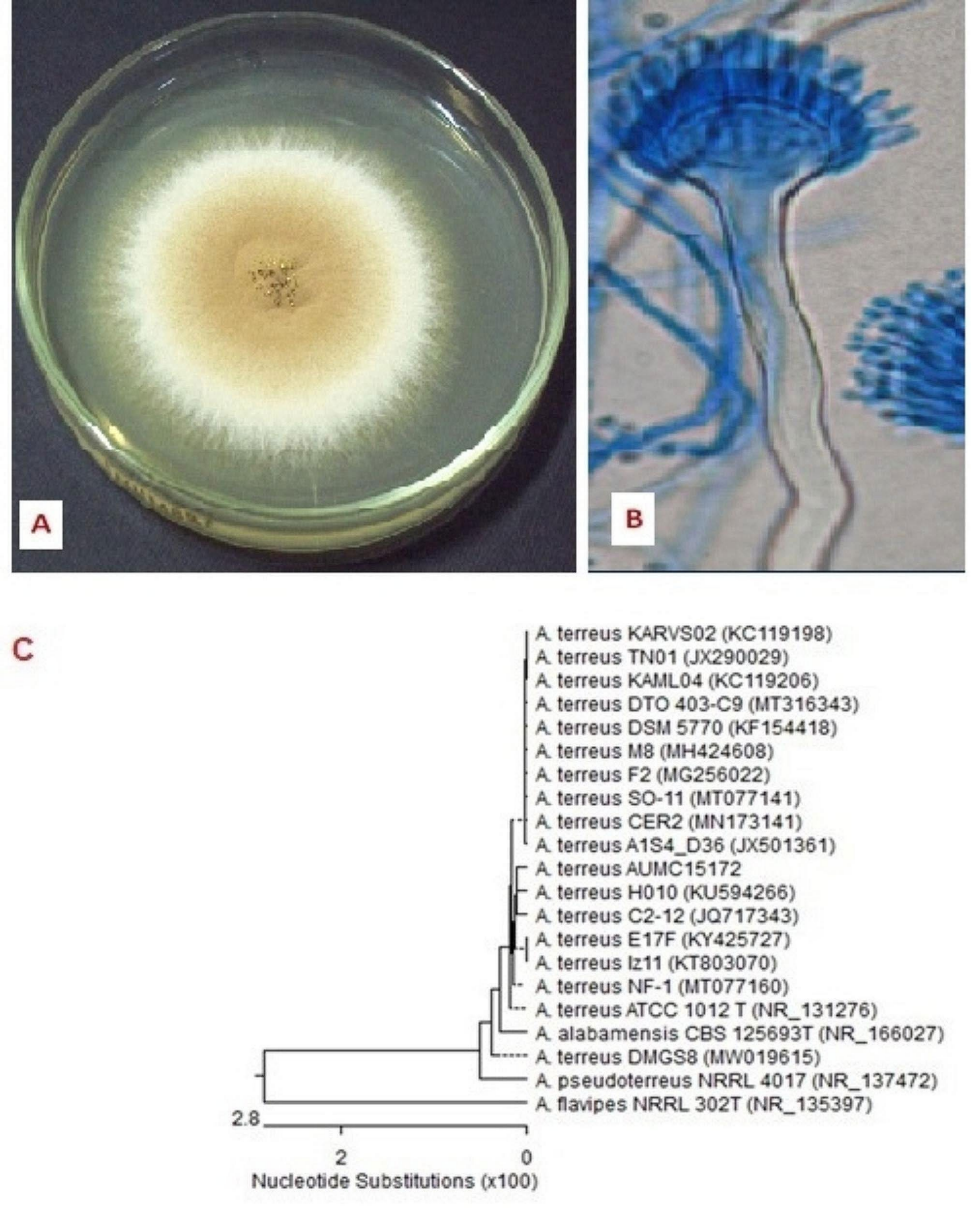
Morphological and molecular characteristics of *A. terreus* AUMC15172. Colony growth was observed on Czapek Yeast autolystae agar after incubation for 10 days at 25 °C (**A**). Microscopic appearance of conidia and conidiophore (**B**). Phylogenetic tree of the fungal isolate (AUMC15172.) and other closely related strains of *A. terreus*, based on the ITS1-5.8 S rRNA-ITS2 rDNA sequences (**C**)

*Aspergillus flavus* AUMC 15,171’s colony morphology is depicted in Fig. 3 as a yellow-green colony after 10 days on Czapek agar. In the meantime, the microscopic view revealed a conidiophore with a rough wall and a radiating, biseriate conidial head with metulae, phialides, and chains of conidia. According to the findings, *A. flavus* AUMC 15,171 displayed 100% coverage and 1005% identification with other strains of *A*. *flavus*. The morphological analysis of the fungus strain cultivated 10 days at 25 °C on CYA agar matched the features used to identify *A. flavus* [29]. Thus, information gleaned from the great degree of congruence with closely related fungi was corroborated by molecular analysis of these strains. *Aspergillus terreus* AUMC 15,172 has a cinnamon-buff to sand-brown colony that is depicted in Fig. 4 as growing on Czapek agar. While conidiophores, columnar biseriate conidial heads, and chains of tiny conidial fragments were visible at a microscopic level. With many strains of *A. terreus*, the sample AUMC15172 displayed 100% identification and 100% coverage. The morphological analysis of the fungus strain kept for 10 days at 25 °C on CYA agar matched the features used to identify *A. terreus* [29]. The high level of congruence with the similarly related fungus was therefore corroborated by information acquired from genetic investigations of these strains.

### Indole acetic acid and siderophores production

Data showed (mention results here without table) that indole acetic acid was generated by three endophytic fungus strains. The strain of *Alternaria alternata* recorded the highest rates from IAA (23.6 µg/ml) than *Aspergillus flavus* and *Aspergillus terreus* strains 21.3 and 20.88 µg/ml respectively. In the literature, previous studies found that the pure culture of endophytic fungi (*Alternaria alternata*, *Aspergillus fumigatus*, *Chaetomium globosum*, *Chrysosporium pseudomerdarium*, *Fusarium* spp., *Paecilomyces* spp., *Penicillium* spp., *Phoma* spp. and *Tulasnella* sp.) could produce IAA after being cultured in liquid medium supplemented with L-Trp [35, 42, 43]. Endophytic fungi produce a variety of secondary metabolites, such as ammonia and plant hormones, especially IAA, that aid in the growth of plants [44]. The capacity of endophyte *F. oxysporum* to colonize maize roots was restored by the use of IAA on an exogenous basis, and many maize seedling growth indices were greatly enhanced [45]. IAA regulates root initiation, cell differentiation, cell division, and cell elongation [46]. Only one out of 27 endophytic fungal isolates obtained from *Coffea arabica* plants in the north of Thailand was examined, according to a study by Numponsak et al. [31], was able to create IAA. The PGP of agricultural crops is significantly influenced by this IAA production capacity. *Alternaria alternata*, which was isolated from the seeds of *Elymus dahuricus*, also boosts photosynthetic capability and enhances nutrient accumulation in plant tissues [47]. *A. alternata* and *F. triticum* both increased stem and root length, as well as chlorophyll content [35]. Additionally, *A. terreus* isolated from paprika plants has the ability to create IAA, which promotes plant development, in vegetable crops like tomato [48].

Regarding the siderophore production, the obtained data confirmed that *Alternaria alternata* AUMC15170, *Aspergillus flavus* AUMC15171, and *Aspergillus terreus* AUMC15172 were negative for siderophore formation on CAS agar against blue medium. Siderophore, a small-molecular-size iron-chelating agent, is created by microbes and plants when there is a lack of iron [42]. Out of 20 maritime mushrooms, only one, *A. flavus*, did not produce siderophores [49]. In general, whether a fungus produces siderophores or not depends on a variety of factors, including environmental conditions, local nutrient concentrations, and the specific physiological and genetic characteristics of the fungus [42, 49].

### Phosphate-solubilizing efficiency

Data obtained that all the endophytes solubilized phosphate. Maximum phosphate solubilization ability was recorded in the case of the fungal isolate AUMC 15,170 (*Alternaria alternata)* being 4.517 µg/ml then AUMC 15,171 *(Aspergillus flavus)* was 1.5 µg/mL. AUMC 15,172 (*Aspergillus terreus)* strains recorded a minimum concentration of 0.4255 µg/ml. Endophytes demonstrate several types of plant growth-promoting effects, including the solubilization of phosphate, the formation of siderophore and IAA, the fixation of nitrogen, the creation of ammonia, and more [50, 51]. According to Junaidi and Bolhassan (2017), different strains of the same fungus can generate varying quantities of metabolites. Fungi that may dissolve phosphates, as noted by Sharma et al. [52] and Alori et al. [53], examples include *Achrothcium*, *Alternaria, Aspergillus, Cephalosporium, Cladosporium, Chaetomium, Fusarium, Glomus, Myrothecium, Penicillium, Phoma, Populospora*, and *Rhizopus*.

### Antioxidant activity and antifungal sensitivity tests

The results of comparing the antioxidant behavior of extracts from three fungal strains to ascorbic acid (Table 2) validated their antioxidant capability. The reported DPPH scavenging values for *A. alternata* AUMC15170, *A. flavus* AUMC1517, and *A. terreus* AUMC15172 were 67.81, 58.55, and 73.49%, respectively. Meanwhile, it was 100% for ascorbic acid. Antioxidant substances are known to have anti-inflammatory, anti-atherosclerotic, anticancer, anticarcinogenic, antibacterial, and antiviral effects [54, 55]. Antioxidants are also beneficial in the management of reactive oxygen species-mediated deficits. In accordance with research by Duan et al. [56] all endophytic fungi contain some level of antioxidant activity. As a result, under stressful circumstances, endophytic *A. flavus* generates new antioxidant metabolites [57] and increases the expression of antioxidant genes [58]. Our results are in line with earlier research on the role of endophytes in facilitating salt tolerance in agricultural plants like tomato [59] and barley [60]. Our findings are consistent with Altaf et al. [61] evaluation of the activity of three of the four endophytes isolated from the medicinal plant. According to the DPPH data, *Aspergillus niger* (88.53%) had the highest level of inhibition, then followed by *Aspergillus flavus* (82.35%), then *Aspergillus terreus* sp (70.1%). Furthermore, an endophytic fungus was isolated from the root of the medicinal plant Moringa oleifera Lam and identified as Nigrospora sp produced four antifungal secondary metabolites [62].

**Table 2:** Antioxidant (% of DPPH scavenging) and antifungal (mm) activities of chloroform extracts of *A. alternata* AUMC15170, *A. flavus* AUMC1517, and *A. terreus* AUMC15172

Fungal extracts	DPPH scavenging (%)	Diameter of inhibition zone (mm)			
		Aspergillus brasiliensis	Botrytis cinerea	Fusarium oxysporum	Penicillium digitatum
*A. alternata* AUMC15170	67.81 ± 11.44^c^	21.67 ± 3.51^b^	19.33 ± 2.52^c^	15.67 ± 2.52^c^	22.67 ± 1.52^c^
*A. flavus* AUMC15171	58.55 ± 10.21^d^	10.67 ± 2.52^c^	13.67 ± 1.53^d^	10.00 ± 3.00^c^	18.33 ± 1.52^c^
*A. terreus* AUMC15172	73.49 ± 14.28^b^	10.33 ± 1.53^c^	25.67 ± 1.53^b^	27.00 ± 2.00^c^	25.33 ± 1.52^d^
Control	100.00 ± 0.00^a^	35.67 ± 3.51^a^	40.00 ± 1.00^a^	25.00 ± 2.00^d^	30.00 ± 2.00^d^

Ascorbic acid (1000 µg mL^− 1^) and nystatin (100 µg mL^− 1^) were used as control antioxidant and antifungal agents, respectively. Calculated mean is for triplicate measurements from two independent experiments ± SD, ^a−f^ means with different superscripts in the same column are considered statistically different (LSD test, *P* ≤ 0.05)

Table 3 presents the antifungal activities of extracts from *A. alternata* AUMC15170, *A. flavus* AUMC1517, and *(A) terreus* AUMC15172 against three plant pathogenic fungi and a human pathogenic fungus. When compared to the conventional antifungal (Nystatin), all extracts showed good antifungal activity. The acquired results (Table 3) demonstrated that the reported values of inhibitory zones differed amongst fungal species. The recorded values of extracts from the respective fungal strains against *Penicillium digitatum* were 22.67, 18.33, and 25.33 mm, against *Fusarium oxysporum* were 15.67, 10.00, and 27.00 mm, against *Botrytis cinerea* were 19.33, 13.67, and 25.67. Moreover, the recorded values of extracts from the respective fungal strains against *Aspergillus brasiliensis* were 21.67, 10.67, and 10.33 mm. Wang et al. [63] conducted an investigation in which 67 species of endophytic fungal isolated from *Quercus variabilis* were dominated by *Aspergillus sp., Penicillium sp., and Alternaria sp.* and considerably showed antimicrobial activity. Similar to this, earlier studies found that *Aspergillus* sp extracts had a modest amount of action against *(B) cinerea*, *F. oxysporum*, and *F. solani* [64].

**Table 3:** GC-MS analysis of the chloroform extract of *Aspergillus terreus* AUMC15172

No.	RT (min)	MW	MF	Area (%)	Detected compounds	Bioactivity	References
**1**	7.26	46	CH_2_O_2_	0.71	Formic acid	Antimicrobial	[101]
**2**	22.41	266	C_18_H_34_O	0.55	4-Octadecenal (spectrum disagrees) (CAS)	Antimicrobial, Anti-inflammatory	[102]
**3**	22.41	238	C_17_H_34_	055	1Heptadecene (CAS)	Anticancer	[76]
**4**	25.58	206	C_16_H_14_	17.96	15-methyltricyclo[6.5.2 (13,14)0.0(7,15)] pentadeca1,3,5,7,9,11,13-heptene	Antioxidant	[76]
**5**	25.58	206	C_14_H_22_O	17.96	2-tert-Butyl 4-isopropyl 5-methylphenol	Antioxidant, antimicrobial	[68]
**6**	25.58	206	C_13_H_18_O_2_	17.96	3,4-Dihydro-2H-1,5-( 3"t-butyl) benzodioxepine	anticancer, antifungal	[87]
**7**	31.78	312	C_20_H_40_O_2_	7.58	Acetic acid noctadecyl ester	Antioxidant, anti-inflammatory	[80]
**8**	31.89	408	C_29_H_60_	1.28	Nonacosane (CAS)	Anti-mutagenic, antibacterial	[66]
**9**	35.82	256	C_17_H_36_O	13.29	1-Heptadecanol (CAS)	Antifungal, antioxidant	[73]
**10**	35.82	266	C_19_H_38_	13.29	9-Nonadecene	Anticancer, antioxidant, antimicrobial	[79]
**11**	35.82	252	C_18_H_36_	13.29	1-Octadecene (CAS)	Anticancer, antioxidant, antimicrobial	[79]
**12**	39.51	354	C_24_H_50_O	6.08	n-Tetracosanol1	Anticancer	[94]
**13**	41.41	254	C_16_H_30_O_2_	0.68	9-Hexadecenoic acid (CAS)	Antimicrobial	[67]
**14**	41.52	536	C_40_H_56_	0.40	Lycopene 7	Antioxidant, anticancer	[103]
**15**	42.90	268	C_18_H_34_D_2_O	2.46	2,2DIDEUTERO OCTADECANAL	Antimicrobial	[104]
**16**	42.90	490	C_35_H_70_	2.46	17-Pentatriacontene (CAS)	anticancer, antibacterial	[105]
**17**	42.90	242	C_16_H_34_O	2.46	1Hexadecanol (CAS)	Antioxidant, Antimicrobial,	[65]
**18**	45.30	390	C_24_H_38_O_4_	5.70	1,2Benzenedicarboxylic acid, bis(2ethylhexyl) ester (CAS)	Anti-microbial	[74]
**19**	47.66	564	C_38_H_76_O_2_	1.36	Octadecanoic acid, eicosyl ester (CAS)	Antimicrobial, Antioxidant	[106]
**20**	47.66	228	C_15_H_32_O	1.36	1Dodecanol, 3,7,11trimethyl(CAS)	Antimicrobial	[107]
**21**	50.21	314	C_21_H_30_O_2_	0.75	Retinoic acid, methyl ester (CAS)		
**22**	51.61	214	C_14_H_30_O	0.84	1Tetradecanol (CAS)	Antibacterial, anti-inflammatory	[83]

### Identification of chemical constituents of the fungal extracts by GC-MS

**Fig. 5:**
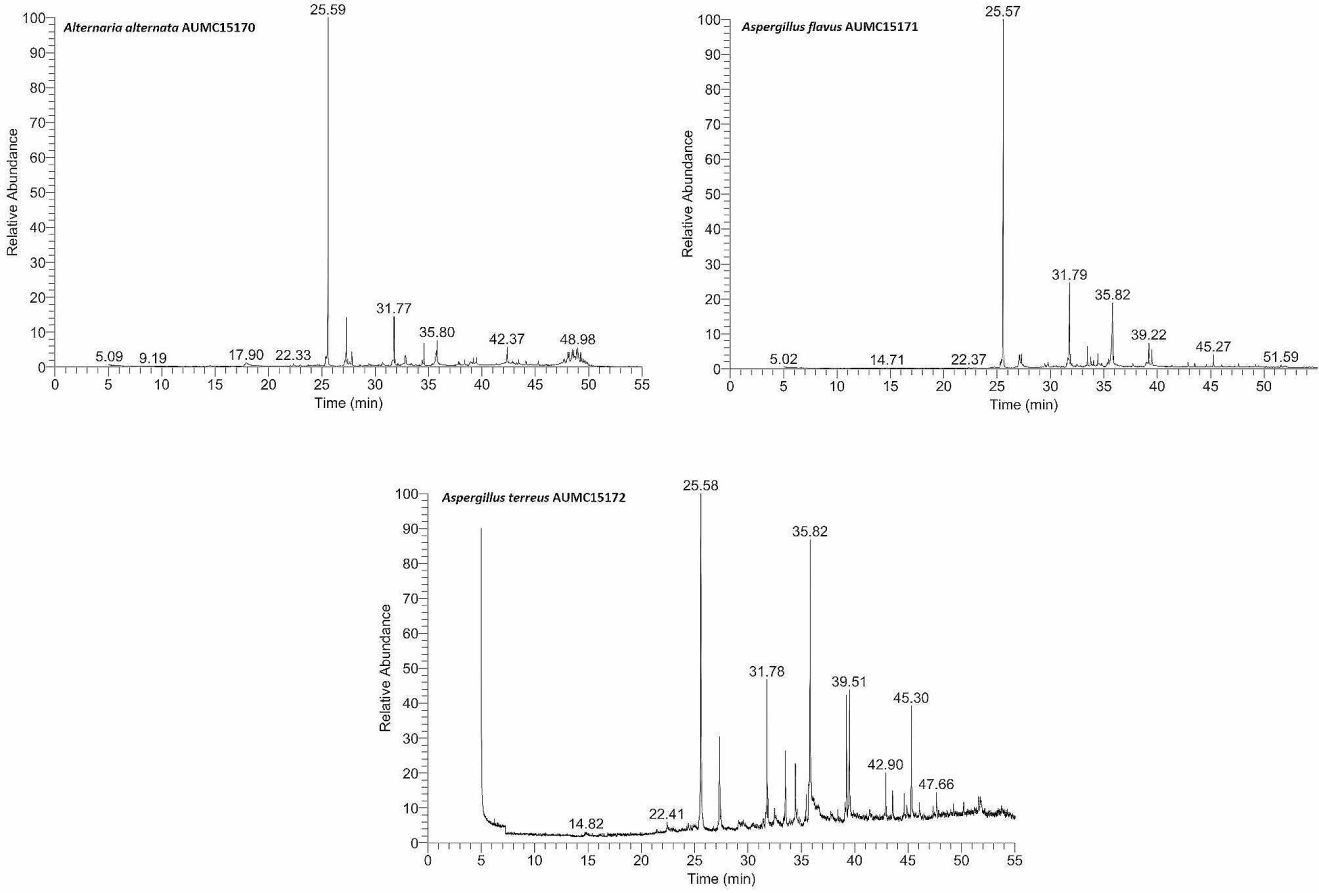
GC-MS chromatograms of crude extracts from the fungal culture of *A. alternata* AUMC15170, *A. flavus* AUMC1517, and *A. terreus* AUMC15172. The chloroform extracts were evaporated and dissolved in 1 mL of HPLC-grade methanol and used under the conditions described in Materials and Methods

The crude extracts of *Alternaria alternata* AUMC15170, *Aspergillus flavus* AUMC15171, and *Aspergillus terreus* AUMC15172 were subjected to GC-MS analysis, which identified a large number of chemicals (Fig. 5). In Tables 4 and 5, and 3 (for *A.alternata* AUMC15170, *A.flavus* AUMC15171, and *A.terreus* AUMC15172), The overall peak area, retention duration, molecular weight, molecular formula, as well as structure of the discovered components in the extracts are all recorded (Fig. 5). The findings supported the existence of various well-known bioactive compounds with different properties, such as antifungal, antibacterial, and antioxidant, and the three fungus strains generated plant growth regulators, in accordance with the literature reported in Tables 4 and 5, and 3.

**Table 4:** GC-MS analysis of the chloroform extract of *Alternaria alternata* AUMC15170

No.	RT (min)	MW	MF	Area (%)	Detected compounds	Bioactivity	References
**1**	17.90	226	C_16_H_34_	1.45	Dodecane, 5,8-diethyl Hexadecane (CAS)	Antifungal, antitumor	[64]
**2**	22.33	242	C_16_H_34_O	0.41	1Hexadecanol (CAS)	Antioxidant, antimicrobial	[65]
**3**	22.33	240	C_16_H_32_O	0.41	Hexadecen-1-ol, trans9	Anti-oxidant, antibacterial	[66]
**4**	22.33	196	C_14_H_28_	0.41	3-Tetradecene, (Z)	Antimicrobial	[67]
**5**	25.59	206	C_14_H_22_O	34.40	2-tert-Butyl-4-isopropyl-5-methylphenol	Antioxidant, antimicrobial	[68]
**6**	25.59	206	C_13_H_18_O_2_	34.40	3,4-Dihydro2H-1,5( 3”-(tbutyl) benzodioxepine	Anticancer, antifungal	[69]
**7**	25.59	206	C_16_H_14_	34.40	15-methyltricyclo[6.5.2 (13,14). 0 (7,15)] pentadeca1,3,5,7,9,11,13- heptene	Antioxidant	[70]
**8**	27.64	222	C_15_H_26_O	0.50	1 H-Benzocyclohepten7-ol, 2,3,4,4a,5,6,7,8- octahy dro-1,1,4a, 7tetramethy -l, cis (CAS)	Antimicrobial, antitumor, antioxidant	[71]
**9**	27.64	222	C_13_H_18_O_3_	0.50	(E,1’R*,2’*,3’*,4’*)4(1’,2’:3’,4’Diepoxy2’, 6’, 6’trimethyl1’ cyclohe xyl)3buten2one	Antimicrobial	[72]
**10**	27.82	222	C_15_H_26_O	2.02	6-epi-shyobunol Or Farnesol	Antimicrobial, antitumor, antioxidant	[71]
**11**	31.77	256	C_17_H_36_O	6.69	n-Heptadecanol-1	Antimalarial, antifungal, Antioxidant	[73]
**12**	31.86	254	C_18_H_38_	0.49	Octadecane (CAS)	Antifungal, antimicrobial	[74]
**13**	31.86	310	C_22_H_46_	0.49	Docosane (CAS)	Antimicrobial, anticarcinoma	[75]
**14**	34.41	276	C_17_H_24_O_3_	0.90	7,9Ditertbutyl1oxaspiro(4,5)deca6,9-diene 2,8-dione	Antimicrobial	[76]
**15**	35.80	298	C_20_H_42_O	2.71	1-Eicosanol (CAS)	Antioxidant	[77]
**16**	35.80	280	C_20_H_40_	2.71	3-Eicosene, (E)	Antimicrobial	[78]
**17**	35.80	266	C_19_H_38_	2.71	9-Nonadecene(CAS)	Anticancer, antimicrobial	[79]
**18**	38.49	312	C_20_H_40_O_2_	1.15	Ethanol, 2(9- octadecenyloxy), (Z)(CAS)	Antioxidant, anti-inflammatory	[80]
**19**	39.49	326	C_22_H_46_O	1.45	1- Docosanol (CAS)	Antiviral, Antimicrobial	[81]
**20**	42.37	266	C_17_H_30_O_2_	2.53	Hexadecadienoic acid, methyl ester (CAS)	Antioxidant	[70]
**21**	48.98	418	C_26_H_42_O_4_	3.80	1,2Benzenedicarboxyli c acid, dinonyl ester (CAS)	Anti-inflammatory, Anti-hypercholesterol	[82]

**Table 5:** GC-MS analysis of the chloroform extract of *Aspergillus flavus* AUMC15171

No.	RT (min)	MW	MF	Area (%)	Detected compounds	Bioactivity	References
**1**	24.72	214	C_14_H_30_O	1.50	1-Tetradecanol (CAS)	Antibacterial, antioxidant	[83]
**2**	24.72	168	C_12_H_24_	0.25	1-Dodecene (CAS)	Antioxidant, antimicrobial	[84]
**3**	25.30	191	C_10_H_6_FNO_2_	0.54	3-Fluoro4-quinolinecarboxylic acid	Antibacterial and antitumor activity.	[85]
**4**	25.57	234	C_15_H_26_N_2_	41.20	1,4,11,11-tetramethyl1,4methanocyclooc ta[d]pyridazine	Antimicrobial, antioxidant, fungicidal	[86]
**5**	25.57	206	C_13_H_18_O_2_	41.20	3,4-Dihydro2H-1,5 (3"tbutyl) benzodioxepine	Anticancer, and antifungal activities.	[87]
**6**	27.09	286	C_14_H_26_N_2_O_4_	2.47	L-Valine, N (N-acetyl L-alanyl), butyl ester	Antimicrobial activity	[88]
**7**	27.09	243	C_8_H_13_N_5_O_2_S	2.47	Butanamide, 2-(1methyltetrazol-5-yl hio)3-oxo N, N dimethyl	Anti-inflammatory, antimicrobial, antioxidant	[89]
**8**	29.70	198	C_14_H_30_	0.32	Tetradecane (CAS)	Antioxidant	[90]
**9**	29.78	220	C_15_H_24_O	0.73	Isoaromadendrene epoxide	Antibacterial	[91]
**10**	29.78	236	C_15_H_24_O_2_	0.73	Murolan3,9(11)diene 10 peroxy	Antifungal	[92]
**11**	30.32	230	C_14_H_30_O_2_	0.25	1-Dodecanol, Ethoxy	Antimicrobial	[93]
**12**	30.32	618	C_44_H_90_	0.25	Tetratetracontane (CAS)	Antioxidant	[66]
**13**	31.78	312	C_20_H_40_O_2_	12.15	2-Hexyldodecyl acetate	Antioxidant, anti-inflammatory	[80]
**14**	31.78	354	C_24_H_50_O	12.15	n-Tetracosanol-1	Anticancer	[94]
**15**	31.78	340	C_22_H_44_O_2_	12.15	1-Dodecanol, 2-octyl, acetate	Antimicrobial, antioxidant	[95]
**16**	31.88	282	C_20_H_42_	0.79	Eicosane (CAS)	Antifungal activity	[96]
**17**	35.82	280	C_20_H_40_	13.44	1Eicosene (CAS)	Antimicrobial activity	[78]
**18**	35.90	296	C_21_H_44_	0.36	Heneicosane (CAS)	Antimicrobial	[97]
**19**	39.21	396	C_26_H_52_O_2_	2.43	Dodecanoic acid, tetradecyl ester (CAS)		
**20**	39.50	312	C_21_H_44_O	1.93	1-Heneicosanol	Antifungal	[98]
**21**	45.28	390	C_24_H_38_O_4_	1.65	1,2Benzenedicarboxylic acid, bis(2ethylhexyl)ester (CAS)	Antimicrobial	[74]
**22**	46.74	354	C_22_H_42_O_3_	0.25	n-Butylricinoleate	Anticancer and antimicrobial	[99]
**23**	49.23	222	C_15_H_26_O	0.38	2,6,10-Dodecatrien-1-o l, 3,7,11-trimethyl	Antimicrobial, antitumor, and antioxidant	[71]
**24**	51.58	330	C_19_H_38_O_4_	0.39	Hexadecanoic acid, 2,3-dihydroxypropyl ester	Pesticide, Antioxidant	[100]
**25**	53.75	346	C_19_H_22_O_6_	0.17	Isochiapin b (Gibberellic acid)	Plant growth regulator	[76]

Generally, the application of endophytes for seed priming has several promising potentials in the field of seed technology and agricultural productivity. Using endophytic fungal cultures and their extracts, certain beneficial compounds could be used by the plant, as supported by our findings. This opens up new avenues for research and development in the field of sustainable agriculture [41, 43]. Our research is in progress to isolate and purify single compounds responsible for the activities and test their applications to some plants.

## Data Availability

The sequence sizes of *Alternaria alternata* (AUMC15170), *Aspergillus terreus* (AUMC15172), *Aspergillus flavus* (AUMC15171) were successfully deposited in NCBI GenBank with accession numbers OR511885 (https://www.ncbi.nlm.nih.gov/nuccore/OR511885), OR511886 (https://www.ncbi.nlm.nih.gov/nuccore/OR511886), and OR511887 (https://www.ncbi.nlm.nih.gov/nuccore/OR511887), respectively.
